# Modulation of the peak velocities and gains of abduction and adduction saccades according to head position

**DOI:** 10.1007/s10384-024-01132-9

**Published:** 2025-01-03

**Authors:** Hana Mino, Hiromasa Sawamura, Koji Takahashi, Hiroya Hara, Yosuke Kudo, Kenzo Yamanaka, Tomoko Kaida, Kazunori Miyata, Makoto Aihara, Ken Johkura

**Affiliations:** 1 Department of Ophthalmology, The University of Tokyo Graduate School of Medicine, 7-3-1, Hongo, Bunkyo-ku, Tokyo, 113-8655 Japan; 2 Department of Ophthalmology, Teikyo University School of Medicine, Tokyo, Japan; 3 Department of Clinical Laboratory, Yokohama Brain and Spine Center, Yokohama, Japan; 4 Department of Neurology, Yokohama Brain and Spine Center, Yokohama, Japan; 5 CREWT Medical Systems Inc., Tokyo, Japan; 6 Miyata Eye Hospital, Miyazaki, Japan

**Keywords:** Saccades, Ocular counter-rolling, Abduction, Adduction, Head tilt

## Abstract

**Purpose:**

To assess the effects of modifying head position and of static ocular counter-rolling (OCR) on abduction and adduction in saccadic eye movements using a head-mounted video-oculographic device.

**Study design:**

A clinical observational study.

**Methods:**

The peak velocities and amplitude gains of visually guided 12° saccades were binocularly measured in 21 healthy volunteers with their heads in the upright vertical (0°) and horizontal (± 90°, bilateral side-lying) postures, and in 6 participants with their head positions bilaterally tilted by 30°. The rotation angles of eyeballs generated via OCR in the bilateral 30° and 90° head positions were evaluated in five participants.

**Results:**

Peak velocities and gains were significantly higher with the head in the 0° position compared to ± 90°. The decreases in peak velocities and gains at ± 90° were not affected by the apogeotropic or geotropic directions. Faster peak velocities and greater gains on abduction, rather than adduction, were observed under each test condition. The tendencies toward faster peak velocity and greater gain in the 0° head position rather than bilaterally tilted at 30° were preserved. The absolute rotation angles at ± 90° were larger than those at 30°.

**Conclusions:**

Head position affected the peak velocities and gains of both abduction and adduction saccades. The findings suggest that modified force vectors exerted by different eye muscles recruited during OCR play a role. Our research provides valuable insights for assessing eye movements across various head positions.

## Introduction

Saccades are rapid eye movements that shift the line of sight from one fixation point to another, supporting vision by directing the fovea of the retina to the point of interest [1–3]. They are commonly evaluated to assess brain function in normal individuals, encompassing motor control, cognition, and memory, and to elucidate the pathophysiological mechanisms in patients with various diseases [2–5]. Moreover, the relationship between head position and saccade status yields useful information when evaluating patients with ocular motor apraxia or degenerative diseases [1]. Thus, it is important to clarify the features of saccades that develop when healthy participants assume different head positions.

When the head is tilted from the normal upright vertical position, adduction and abduction eye movements are no longer horizontally aligned with the earth. Such directional changes may affect the characteristics of eye movements. Changes in gravity trigger reflective eye torsion, known as ocular counter-rolling (OCR), a compensatory anti-directional torsional eye movement around the visual axis that is opposite to the axis of lateral head tilting [6–9]. Two types of OCR are described: dynamic OCR during active head roll movement and static OCR during static head tilting [7]. The latter is believed to reflect otolith stimulation when the static head position is influenced by the direction of the gravitational force [10, 11]. Therefore, saccades associated with different head positions may be influenced by OCR. Indeed, the trajectories of both horizontal and vertical saccades are modulated by static OCR [12].

Recent advancements in technology have made video-oculography a viable tool for assessing eye movements [13, 14]. This study explored whether the peak velocities and gains of abduction and adduction saccades differ according to head position. Additionally, we evaluated the effects of OCR on these properties using a head-mounted video-oculography system.

## Subjects and methods

### Study design and participants

This was a clinical observational study. The study protocol was approved by the Ethics Committee of the University of Tokyo Hospital. All participants provided written informed consent in accordance with the tenets of the Declaration of Helsinki.

Twenty-one healthy participants (mean ± SD age: 26.9 ± 2.0 years; 12 men and 9 women) participated in the study. All participants were healthy and had no histories of neurological, otolaryngological, or ophthalmological disorders. All participants exhibited normal or corrected-to-normal visual acuities OU.

### Equipment

A head-mounted eye movement-recording device (imoHE; CREWT Medical Systems) was used. The imoHE, a new dedicated video-oculography device, replaces the original imo head-mounted device that evaluated perimetric parameters [15–17]. This device incorporates a CMOS sensor camera (XIMEA) operating at 300 Hz for recording pupil images [18]; it is also equipped with independent optical systems that show the visual targets and tracking systems of each eye [16, 18]. The procedures for correcting participant spherical power, focusing of the pupil image, and adjusting the pupil position to the center of the monitor when using the imoHE were identical to the procedures utilized with the original imo, as detailed elsewhere [15].

**Fig. 1:**
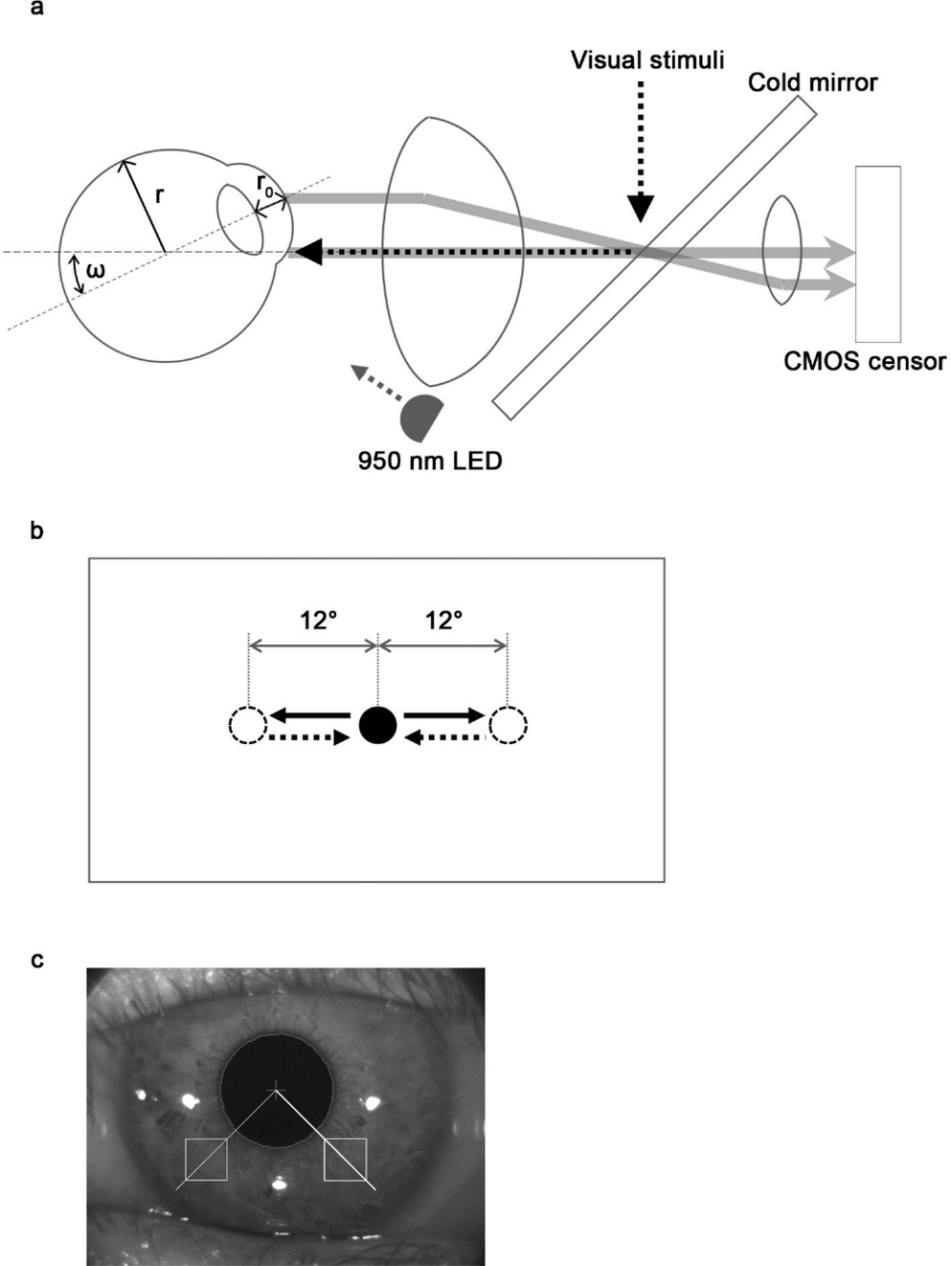
**a** A schematic representation of an optical system as observed by the imoHE. “r” indicates the eyeball radius; “r_0_” the distance between the corneal apex and the pupil center; and “ω” the visual angle calculated by the imoHE. **b** A schematic of the visually guided saccades task. The visual target appears in the center or within 12° of the center. The black solid and dashed arrows indicate the directions of saccades from the center toward the peripheral target points, or vice versa, respectively. Only trials with eyes directed to the peripheral point (black solid arrow) were analyzed. **c** A sample iris image used during the calculation of eye rotation angles. The two white squares on the iris were used to monitor and track the iris patterns. The two squares bracketed the center of the pupil, and both white squares moved as the iris rotated

The imoHE calculates the visual angle of the eye based on the central pupil position detected using a pupil contour of the corrected image and a corneal light-reflection spot estimated by three near-infrared rays (Fig. 1a). The equation for calculation of the visual angle is as follows:

$$\:\omega\:={\text{sin}}^{-1}\left(\text{m}\times\:{\text{m}}_{\text{0}}\times\:\varDelta\:/\left(\text{r}-{\text{r}}_{\text{0}}\right)\right),$$

where m is magnification of the optical system, m_0_ is magnification by the cornea, r is the radius of the eyeball, r_0_ is the distance between the corneal apex and the central pupil position, and Δ is the relative disparity between the pupil central position and the corneal light-reflection spot.

The imoHE was secured on a participant’s head with a belt around the head and hard cushions surrounding the orbit, the wing of the nose, and the temporal bone, thereby maintaining the relative position of the face and the imoHE unchanged during eye movement measurements. Additionally, the imoHE is equipped with a gravitational acceleration sensor for real-time three-dimensional monitoring of the head position [19].

### Behavioral procedures

#### Visually guided saccades

**Fig. 2:**
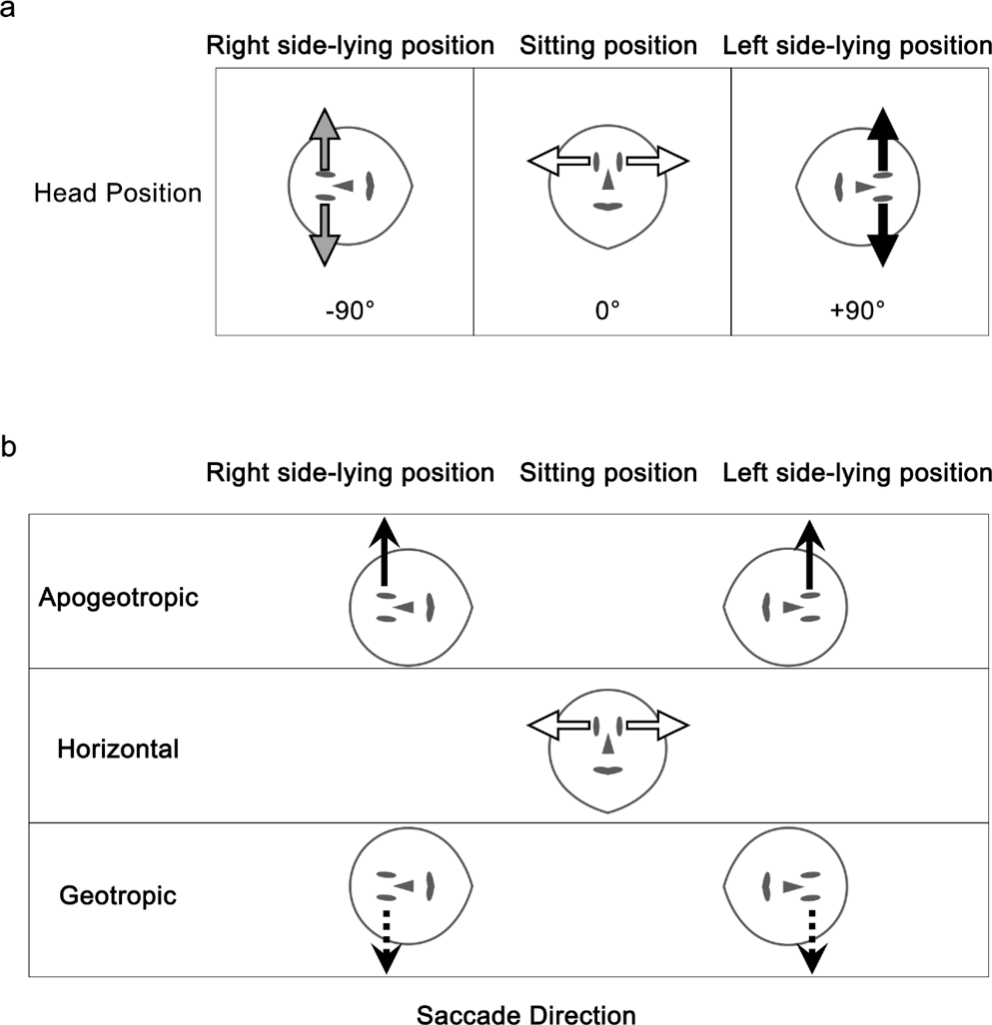
Illustrations of three different head positions and saccade directions, showing only abduction examples. **a** Three head positions: right side-lying, sitting and left side-lying, corresponding to -90°, 0° and 90°. Gray, white, and black solid arrows indicate the directions of abduction at -90°, 0° and 90°, respectively. **b** Apogeotropic, horizontal, and geotropic direction of saccades. Solid black arrows, white arrows, and dashed black arrows indicate the direction of apogeotropic, horizontal, and geotropic saccade, respectively

We used a visually guided saccades task. A black circular visual target (luminance: 0.318 cd/m^2^, Goldmann size: III, visual angle: 0.431°) was simultaneously presented to both eyes against a gray background (luminance: 31.8 cd/m^2^ [100 asb]) at an optical distance of 1 m on an independent 0.7-inch LCD monitor (resolution: 1,920 × 1,080 pixels; refresh rate: 60 Hz). The visual target was initially presented at the center of the display, then jumped either to the right or left by 12°, and subsequently jumped back to the center of the display. This sequence occurred until the visual target appeared nine times on each side in a pseudo-random order, for random durations ranging from 700 ms to 1,300 ms (Fig. 1b). Each participant was required to generate a saccade to the visual target as accurately as possible. Thus, each block of trials consisted of 36 saccades. The visually guided saccades task was conducted with the participant in three postures (sitting upright, and lying on the right and left sides), associated with three different head positions: one vertical (0°) and two horizontal (90°) (Fig. 2a). During measurements in the upright sitting posture, the imoHE was vertically affixed to the table with an attachment. For measurements in side-lying postures, the participants were required to lie on their right or left side on a bed, with the imoHE placed on their head. The imoHE’s position was monitored using the gravitational acceleration sensor and adjusted as necessary. The order of these postures was randomly modified for each participant. Additionally, the visually guided saccades task was performed at two different head positions—30° tilt to the right or left—in six participants. In this scenario, the imoHE was affixed to the table and tilted 30° to the appropriate side.

#### Ocular counter-rolling

The rotation angles of static OCR generated by varying the head position from vertical (0°) to horizontal (i.e., either 90° to the right or left in the side-lying postures) and from vertical to either 30° to the right or left were evaluated in five participants. In a trial under the former condition, the participant put on the imoHE and sat on the bed in an upright posture for 3 s, then slowly bent the body to lie on the right or left side, and remained in the lying position for at least 2.5 s. During this process, monitoring was conducted using the gravitational acceleration sensor, and the imoHE was adjusted to the correct position. In a trial under the latter condition, the participant put on the imoHE, which was fastened to the table with an attachment, and the experimenter tipped the imoHE with a handle until it inclined 30° to the right or left. In both conditions, the participant was instructed to focus on the black central fixation point presented in the bilateral displays, and iris images were recorded. Rotation in the anti-clockwise direction from the participant’s viewpoint was defined as positive when evaluating rotation angles.

### Data analysis

During the visually guided saccades tasks, the visual angles of each eye as a function of time were subjected to off-line analysis. To exclude any effects of prediction, only the saccade trials involving movement toward the peripheral target from the central position were evaluated (Fig. 1b). The disparities in visual angles between two adjacent points of the respective measuring points were calculated as slopes over 18 trials. The beginning and end of a saccade were defined based on velocity criteria; thus, more than 40°/s for the beginning of a saccade and less than 30°/s for the end of a saccade [20]. To eliminate trials that included blinks, or trials affected by image processing errors during off-line analysis, trials exhibiting differences of more than 100°/s in peak velocity between the eyes within a trial, and trials with left/right eye ratio gains less than 50% or more than 200% within a trial, were excluded from analysis, based on the sporadic nature of saccades (conjugate eye movements). Peak velocity was defined as the maximum positive slope between the beginning and end of the saccades. Gain was defined as the percentage of the visual angle at the end of the saccades divided by 12. Saccade latency was not evaluated because the device did not permit this; two independent timers were used to present visual stimuli and to video-record eye movements. To test the symmetry of abduction and adduction during each saccade trial, the peak abduction and adduction velocity and gain of each saccade (i.e., abduction of the right eye and adduction of the left eye, or vice versa) in the vertical head position were compared using paired t-tests. Then, the peak velocities and gains of all trials were averaged for each participant and analyzed using a linear mixed model with the participants regarded as random effects; we utilized JMP Pro 17.0 software. First, the fixed effects of the head positions on the peak velocity and gain of the two eye movements were scrutinized. Second, the fixed effects of the directions (relative to the earth) of the saccades on the peak velocities and gains of the two eye movements were assessed. The trials were classified into three groups based on the head positions and the directions of the saccades: the horizontal direction in the upright sitting posture, and the apogeotropic and geotropic directions in the side-lying postures (Fig. 2b).

In terms of OCR, off-line analysis of iris pattern images was used to calculate the rotation angles [19]. Two 45 pixels × 45 pixels squares were located on the iris image to the right and left of the center of the pupil and below the pupil, one-tenth the diameter of the pupil from the pupillary edge (Fig. 1c); these two squares were tracked during the trial. An iris image obtained immediately before head movement served as a reference. Iris images taken 0.5 s after a participant reached the desired side-lying postures (± 90°) or the ± 30° head tilt positions were used for analysis. The deviations of the two squares in consecutively analyzed images (compared with the reference image) from the center of the pupil were averaged and defined as the rotation angles. Data from two trials at ± 90° and ± 30° head positions were obtained and averaged for each participant.

## Results

**Fig. 3:**
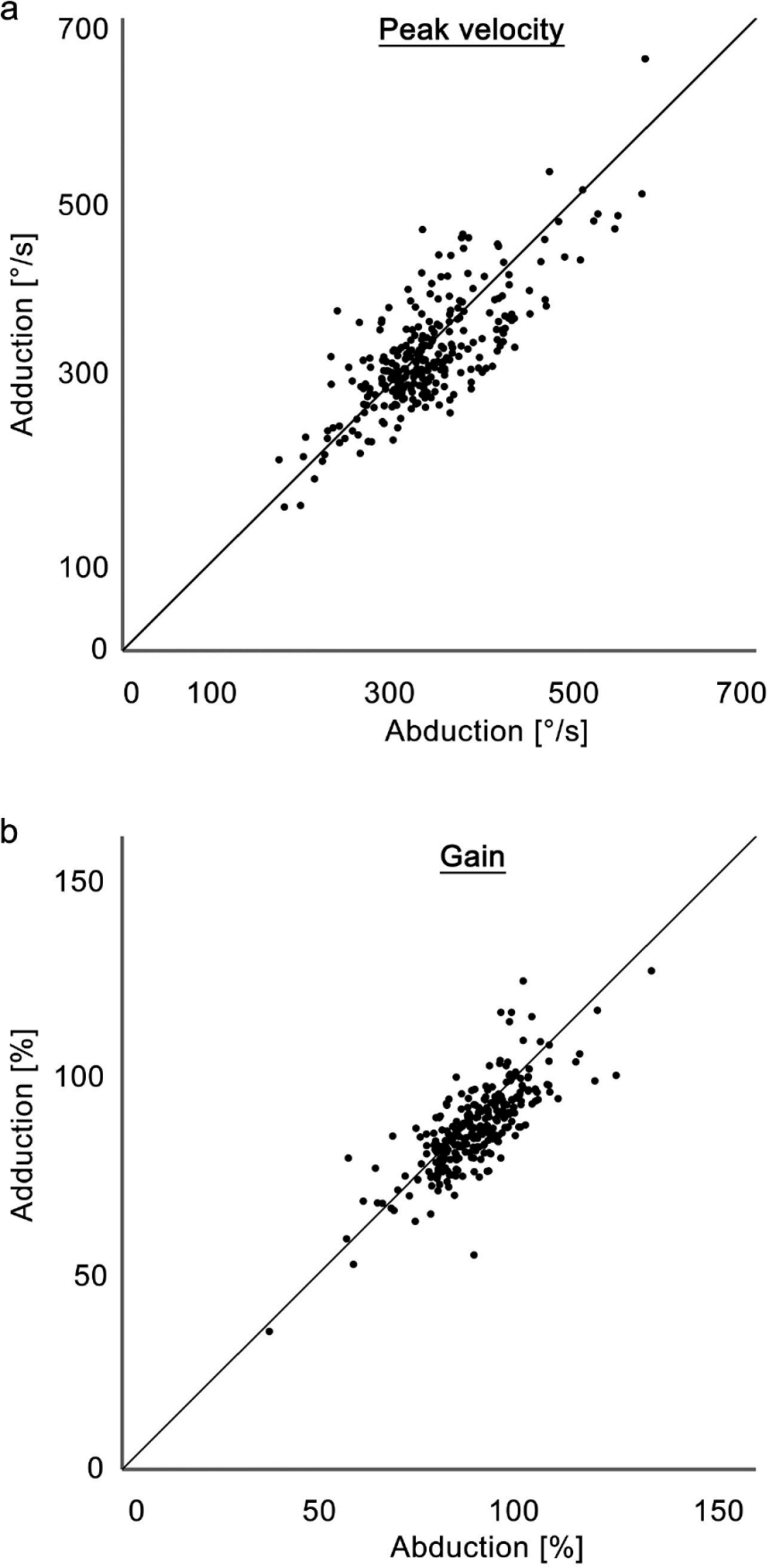
Scatter diagrams of the peak velocities (**a**) and gains (**b**) for abduction (abscissa) and adduction (ordinate) when the head was vertical. Each point corresponds to data from an individual trial. The diagonal line indicates the region where abduction and adduction were equivalent

The peak velocities and gains during abduction and adduction with the head vertical were compared by drawing scatter diagrams (Fig. 3). In these plots, each dot corresponds to a saccade trial. The proportion of trials below the diagonal was larger than above the diagonal (59.4% in terms of peak velocity and 68.0% in terms of gain); paired t-tests revealed that the peak velocity was faster (*t* = 3.76, *df* = 277, *p* =.0002) and the gain was higher (*t* = 6.81, *df* = 277, *p* ≤.0001) during abduction than during adduction.

**Fig. 4:**
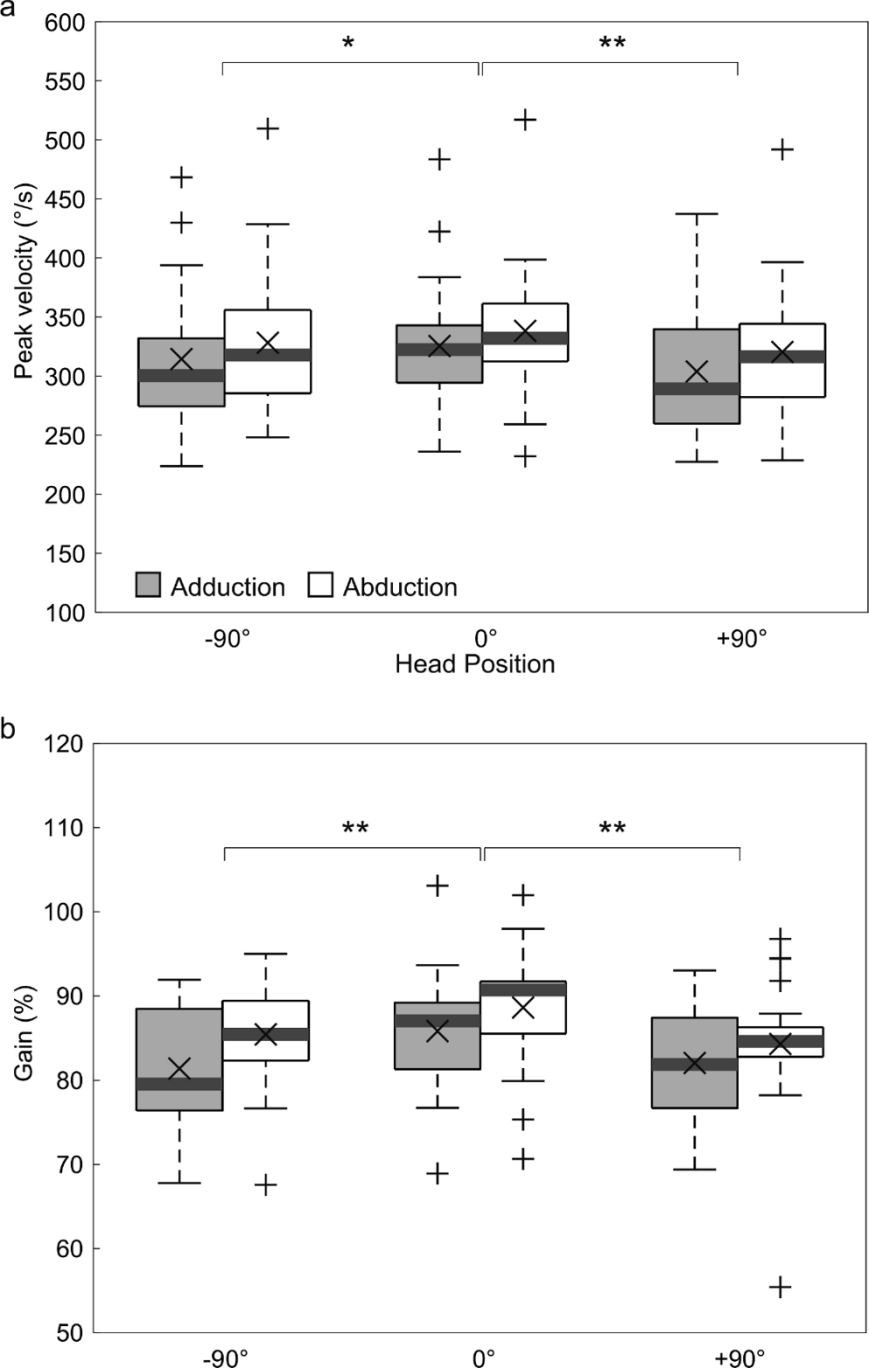
Box plots of the peak velocities (**a**) and gains (**b**) for six combinations of two eye movements (abduction and adduction) and three head positions (0° and ± 90°). The white and gray boxes represent the results for abduction and adduction, respectively. The bold gray lines indicate the medians, the X values represent means, and the top and bottom edges of the boxes are the 75th and 25th percentiles, respectively. The crosses are outliers, and the whiskers indicate the furthest data points (excluding outliers). The asterisks indicate levels of statistical significance as revealed by linear mixed model analyses (*p <.05, **p <.01)

The peak velocities and gains of abduction and adduction at different head positions are shown in Fig. 4a and b. A linear mixed model analysis that considered the two eye movements (abduction and adduction) and the three head positions (0° and ± 90°) as fixed effects revealed significant variances in mean peak velocity and gain between the two eye movements, indicating faster peak velocity and higher gain during abduction (*F*(1, 102) = 15.8, *p* <.01 for the peak velocity and *F*(1, 102) = 12.5, *p* <.01 for the gain), as well as significant variance among the three different head positions (*F*(2, 102) = 10.0, *p* <.01 for the peak velocity and *F*(2, 102) = 9.4, *p* <.01 for the gain). Further post hoc testing revealed that the mean peak velocity and gain were greater in the vertical (0°) head position than in the horizontal (± 90°) head positions (*Tukey test*,* p* <.05).

**Fig. 5:**
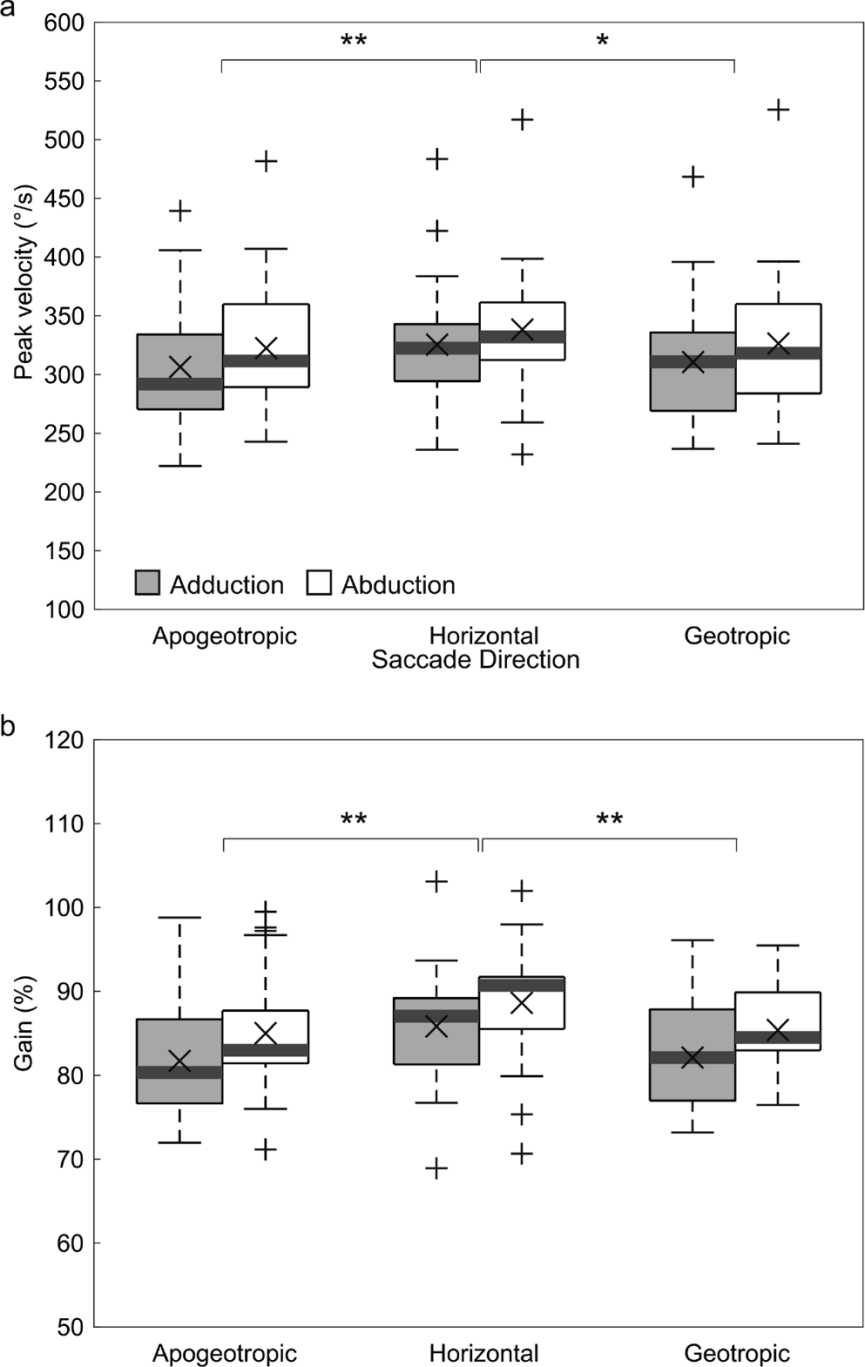
Box plots of the peak velocities (**a**) and gains (**b**) for six combinations of two eye movements (abduction and adduction) and three directions (horizontal, apogeotropic, and geotropic). Acronyms are defined in the legend for Fig. 4

The effects of the directions of the saccades were also assessed. The peak velocities and gains in the various saccade directions (i.e., horizontal, apogeotropic, and geotropic) are shown in Fig. 5a and b. A linear mixed model analysis that considered the two eye movements and the three saccade directions as fixed effects also revealed significant variances in the means of peak velocity and gain, which were greater during abduction (*F*(1, 102) = 16.0, *p* <.01 for the peak velocity and *F*(1, 102) = 14.0, *p* <.01 for the gain), along with significant variance among the three different saccade directions (*F*(2, 102) = 8.0, *p* <.01 for the peak velocity and *F*(2, 102) = 8.7, *p* <.01 for the gain). Further post hoc Tukey testing revealed that mean peak velocities and gains were higher in the horizontal direction than in the vertical, apogeotropic, and geotropic directions (*p* <.05).

Differences in the peak velocities and gains among the three different head positions were apparent, but not when geotropic or apogeotropic saccades were compared. To assess whether differences were evident with slight head tilt, the peak velocities and gains at the two different head positions of ± 30° were evaluated in six participants. The means and standard errors of the peak velocities and gains are listed in Table 1. Although statistical significance was not attained considering the small number of participants, the peak velocity and gain in the vertical position were, on average, greater than those values in the head-tilted positions.

**Table 1:** Peak velocities and gains (means ± SEs) at head positions of 0° and ± 30° (*n* = 6)

		-30°	0°	+ 30°
Peak velocity (°/s)	Abduction	322.3 ± 31.6	331.8 ± 35.9	326.8 ± 33.8
	Adduction	309.9 ± 26.7	320.9 ± 31.8	305.2 ± 29.1
Gain (%)	Abduction	84.2 ± 1.8	85.8 ± 3.5	82.5 ± 1.7
	Adduction	81.3 ± 2.2	83.4 ± 2.3	79.1 ± 1.5

*SE* standard error

The rotation angle of the static OCR in the side-lying postures (± 90°) and the ± 30° head-tilted positions averaged for five participants are listed in Table 2. The absolute rotation angle did not differ between the eyes or between the directions of the head positions at both ± 30° and ± 90°. The absolute rotation angle at a head position of ± 90° was more than double the angle at ± 30°.

**Table 2:** Absolute rotation angles (mean ± SD, °) of static OCR at head positions ± 30° and ± 90° (*n* = 5)

	-30°	+ 30°	-90°	+ 90°
Right eye (°)	2.2 ± 1.3	-2.2 ± 0.9	5.4 ± 2.8	-5.8 ± 1.6
Left eye (°)	1.9 ± 1.0	-2.3 ± 0.9	5.1 ± 1.8	-5.1 ± 1.5

*SD* standard deviation

*OCR* ocular counter-rolling

## Discussion

We found that the peak velocities and gains differed among the three head positions (i.e., the vertical and two horizontal head positions). The peak velocity was faster and the gain was higher in the vertical head position than in the horizontal head positions.

The mean peak velocity observed in the current experiment was consistent with velocities in reports that assessed the peak velocity and gain during modifications of the amplitude from 1.25° to 80° or from 5° to 15°; these values were approximately 340°/s at an amplitude of 10° [21, 22]. The mean gain in the current experiments reflected undershooting of the target, a phenomenon commonly observed in healthy participants [1, 23, 24].

We observed faster peak velocity and higher gain during abduction than during adduction. This difference was consistent regardless of the head position, indicating that the difference was not attributable to head position. These results are not consistent with our previous report, which showed faster peak velocity and higher gain during adduction [13]. One explanation for this discrepancy is that the present experimental conditions differed from the previous work. The distance between the eye and visual target was smaller, and we obtained binocular recordings of the eye movements. Moreover, all participants viewed visual stimuli presented by independent optical systems in front of the participants, which may have influenced accommodative convergence. Indeed, abduction/adduction asymmetry during saccades has differed among reports using various experimental approaches. Some studies reveal faster peak velocity during abduction [21, 25] or adduction [13, 26]; other studies show no difference between the two conditions [27].

We observed faster peak velocities and higher gains for both the abduction and adduction saccades when head position was vertical rather than horizontal. Moreover, we noted no differences in peak velocities or gains between the toward-gravity (geotropic condition) and against-gravity (apogeotropic condition) directions, but we observed faster peak velocities and higher gains at right angles to the gravitational direction (horizontal conditions). The absence of any difference in terms of saccade velocity and gain between the apogeotropic and geotropic conditions suggest that the decreases in peak velocities and gains recorded in lateral positions were not attributable to any direct effect of gravity on the mass of the eyeball or its appendages. In contrast, the rotation angles around the vertical head position increased according to the extent of head tilt (30° and 90°) (Table 2). This finding is consistent with a report showing that the rotation angle increased according to head roll angle, up to approximately 60°; it attained a maximum of 4.9° on average [10]. Thus, OCR generated by head tilting may have affected the peak velocity and gain. One possible explanation is that the extraocular muscles recruited to perform abduction and adduction varied among head positions. When eyes experience torsional deviations, the force vectors of each rectus muscle change; such decomposition of the vectors may reduce the involvement of lateral or medial rectus muscles but increase the involvement of superior and inferior rectus muscles in the directions of abduction and adduction [28]. Indeed, shifts of the rectus pulley in response to head tilting, and thus static OCR, have been observed on magnetic resonance imaging [29]. Our hypothesis is also supported by the observation that the trajectories of vertical and horizontal saccades were modulated by static OCR elicited by rolling the whole body 45° to the right and left, although statistical significance was not observed when horizontal saccades (i.e., abduction and adduction) were examined [12]. In a previous report, dynamic interactions of the eye muscles were suspected to contribute to vertical saccades, as were shifts in eye muscle pulleys [12]. This is consistent with our observations; even small static OCR (30° of head tilt) reduced the mean peak velocity (Table 1).

In this experiment, we assessed saccades using a predefined, black, circular visual target, focusing on differences in saccade gains and peak velocities across head positions. Previous studies show that a larger target size results in faster peak velocity and lower gain with variable saccades endpoints, and that the peak velocity varies with different visual target images [20, 30, 31]. Therefore, modifying target size or shape in relation to head positions may be a subject for future research.

Eye movement tracking devices are already in use across various research fields [32] and have potential as biomarkers for conditions such as hemi-spatial neglect following acute supra-tentorial stroke or subclinical oculomotor disorders in multiple sclerosis [33–35]. As quantitative evaluation of eye movements in various head positions become more common, our findings could have significant clinical implications.

This study had some limitations. First, we evaluated peak velocity and amplitude gains but not saccade latency. Peak velocity and amplitude reflect changes in the motion characteristics of the saccade according to head position, thus the direction of gravity and the resulting OCR, whereas latency primarily depends on central nervous system initiation of saccades. This study focused on changes in eye movement characteristics’ according to head position. However, it may be necessary to measure latency in the future; such measurements would be aided by improvements to the imoHE. Second, we tested only specific head positions. Evaluating saccades in a broader range of head positions could clarify their influence. To confirm the effect of ocular torsion, a detailed comparison of the abduction and adduction saccades with the head in a slightly tilted position is required, as well as an analysis of slightly upward or downward oblique abduction and adduction saccades with the head in an upright position. Third, because we did not utilize electromyography, the detailed effects on the forces exerted by the extraocular muscles remain unclear. Finally, our evaluations involved a relatively small number of subjects, which may limit the generalizability of our findings, although we used intra-subject comparisons rather than inter-subject comparisons.

In conclusion, our findings demonstrate that head position modulated the peak velocity and gain during both abduction and adduction saccades; we utilized head-mounted video-oculography. This work suggests that static OCR contributes to changes in the force vectors of the extraocular muscles. Our findings add information that will aid the assessment of eye movements when a subject’s posture is not upright. Moreover, our technique can be applied to the noninvasive evaluation of otolith function [36].
